# Impact of soil inorganic nitrogen on bacterial phylogeny in estuarine intertidal zones: a study of nitrogen metabolism

**DOI:** 10.3389/fmicb.2023.1341564

**Published:** 2024-01-05

**Authors:** Siqi Li, Tianyang Liu, Cheng Liu, Donglei Sun, Qin Yan, Dengzhou Gao, Zongxiao Zhang

**Affiliations:** ^1^Department of Military Oceanography and Hydrography and Cartography, Dalian Naval Academy, Dalian, China; ^2^Shandong Key Laboratory of Eco-Environmental Science for the Yellow River Delta, Binzhou University, Binzhou, Shandong, China; ^3^Key Laboratory of Humid Subtropical Eco-geographical Process of Ministry of Education, College of Geographical Sciences, Fujian Normal University, Fuzhou, China; ^4^School of Environmental Science and Engineering, Southern University of Science and Technology, Shenzhen, Guangdong, China

**Keywords:** estuarine accommodation space, soil inorganic nitrogen, bacterial phylogenetic characterization, nitrogen metabolism characteristics, environmental response

## Abstract

Here we investigated the potential impacts of soil inorganic nitrogen (SIN) content on the phylogenetic characteristics and ecological functions of soil bacterial communities in estuarine intertidal zones in China, aiming to comprehend the response mechanism of soil microorganisms to variations in SIN content within estuarine wetlands. Our results show that SIN in estuarine areas has a significant spatiotemporal variation on spatial and seasonal scales, in this study and is significantly associated with the phylogenetic diversity and phylogenetic turnover of soil bacterial communities. In addition, the results of the metagenomic analysis showed that the relative abundance of nitrogen-cycling functional genes in bacterial communities did not differ significantly in sampling sites and seasons, and weakly correlated with SIN content. Further, the results based on structural equation modeling (SEM) analysis showed that SIN directly and significantly regulated the phylogenetic characteristics of bacterial communities, thereby indirectly affecting the potential of bacterial nitrogen metabolism. This study emphasizes the key influence of SIN variations on the phylogenetic dissimilarity in soil bacterial communities. Moreover, although there was a weak direct relationship between the functional characteristics of the bacterial nitrogen metabolism and SIN content, the spatiotemporal variation of bacterial nitrogen metabolic potential may be indirectly regulated by SIN content by influencing the phylogenetic diversity in bacterial communities. Our study unravels the pivotal mechanisms through which SIN content influences bacterial communities, thereby offering novel insights into the microbial intricacies governing nitrogen metabolism within estuaries.

## 1 Introduction

Nitrogen (N) transformation in the estuarine soil environment is a hot topic in the study of the estuarine ecological environment (Schmadel et al., 2018). As a key area where rivers and oceans meet, various geochemical and biological factors in estuarine ecosystems change dramatically, significantly affecting the nitrogen transformation process in sediments (Baker et al., 2015). Due to the excessive use of nitrogen fertilizers in agricultural production and the burning of fossil fuels in industrial production, the nitrogen produced by anthropogenic endeavors has increased significantly, and the amount of reactive nitrogen produced by human activities in China will reach 63 Tg by 2050 (Erisman et al., 2008; Galloway et al., 2014), causing a series of environmental problems. Excess inorganic nitrogen is transported to estuaries and coastal zones through groundwater, rivers, and the atmosphere, thus further aggravating the nitrogen load in soils in this area, leading to a range of environmental problems, such as red tide, hypoxia, excessive greenhouse gas emissions, and so on (Diaz and Rosenberg, 2008; Hou et al., 2013, 2015). Estuarine intertidal wetlands play a crucial role in mitigating excessive reactive nitrogen load within marine environments (Pennings, 2012). Nitrogen plays a vital role in microbial cycling and energy metabolism, as well as being essential for the biosynthesis of cellular components like proteins and nucleic acids. In the atmosphere, nitrogen serves as an important reservoir for natural microorganisms; however, only certain archaea and nitrogen-fixing bacteria can directly utilize it. Other microorganisms rely on more active forms of nitrogen, such as ammonium and nitrates, for their material metabolism. The availability of this growth-limiting nutrient is primarily regulated by microbial reactions that modify the REDOX state of nitrogen (Kuypers et al., 2018).

Bacteria are key groups that mediate nitrogen transport and transformation in estuarine sediments, apart from ammonia-oxidizing archaea, most microorganisms involved in nitrogen cycling belong to bacteria (Baker et al., 2015). These bacteria participate in nitrogen transformation and cycling through various pathways, including nitrifying bacteria, denitrifying bacteria, and nitrogen-reducing bacteria. In turn, nitrogen content in soils also has a significant impact on bacterial communities (Gomez-Velez et al., 2015; Jiang et al., 2019; Li et al., 2019). The effects of soil physiochemical characteristics on nitrogen transformation have been systematically discussed, but the important role of the microbial community is easily ignored, and due to the complexity of the microbial community, the relationship between microbial community and soil inorganic nitrogen (SIN) content lacks in-depth research (Schmadel et al., 2018).

Phylogenetic characteristics of microbial communities are the result of long-term environmental adaptation, which provides a new perspective for understanding nitrogen metabolism processes driven by microbial communities (Koskella et al., 2017; Gao et al., 2021). Based on contemporary microbial evolutionary theory, microbial community composition and diversity in the environment are affected by community assembly processes, drawing on the characteristics of phylogenetic turnover (Yuan et al., 2021). However, current research often focuses on the relationship between microbial ecological characteristics and microbial community composition’s temporal and spatial variation and turnover (Stegen et al., 2013; Jiao et al., 2019). Although some studies have verified the positive correlation between the microbial community assembly processes and soil carbon chemical composition, and confirmed the relationship between microbial ecological process and geochemical function (Stegen et al., 2016, 2018a,b), the relationship between phylogenetic turnover of the estuarine microbial community and soil nitrogen concentration in sediments is still lacking.

Estuarine surface soil is the most active area of geochemical processes in estuarine regions, and bacterial communities are the most abundant and functional microbial groups. Based on this, this study focuses on the potential relationship between nitrogen content in the surface soil of coastal zones in China, and the ecological characteristics of the bacterial community construction process, to provide new insights for the study of geochemical cycles in estuarine regions. Specifically, the following three points are included: 1. Are the phylogenetic characteristics of bacterial communities in estuarine sediments regulated by SIN content? 2. Does the SIN content in estuarine sediments affect the nitrogen metabolism function of bacterial communities? 3. What is the potential relationship between the phylogenetic characteristics of bacterial communities and their nitrogen-cycling functional profiles?

## 2 Materials and methods

### 2.1 Soil sample collection

Soil samples were collected from 10 coastal location in China, ranging from high-latitude sites (S1 to S5, latitude >32°) to low-latitude sites (S6 to S10, latitude <32°). 3 to 5 sampling areas were selected from each estuary area, and 15 to 20 soil samples were collected and uniformly mixed as representative samples of one sample point. A total of 78 surface soil samples (0–5 cm) were collected, and 39 surface soil samples were selected in March and September 2019, respectively (Supplementary Table S1). Subsequently, the samples were put into sterile bags, and one part of the sediments was stored in liquid nitrogen for subsequent DNA extraction, and the other part was stored at −20°C for relevant soil property analysis.

### 2.2 Soil environmental properties and inorganic nitrogen content

Standard test methods were adopted to measure soil pH, moisture, cation exchange capacity, organic matter, dissolved organic carbon, microbially oxidizable ferrous iron (Fe^2+^), reducible ferric iron (Fe^3+^), sulfide concentrations, and SIN in each sample, including exchangeable nitrite (NO_2_^−^-N), nitrate (NO_3_^−^-N), ammonium (NH_4_^+^-N). In addition, using the world climate database^1^ to obtain the climate characteristics of each sampling point, including annual mean temperature (MAT) and annual mean precipitation (MAP). The specific analysis methods and soil properties in these collected samples have been given by the previous research (Zhang et al., 2023).

### 2.3 Analysis of bacterial community phylogenetic characteristics and nitrogen-cycle pathway

Sequence analysis was the same as before research methods (Zhang et al., 2023). Briefly, sequences were split into operational taxonomic units (OTUs) at a 3% dissimilarity level using UPARSE, OTUs with less than 2 sequences were removed, and the OTU representative sequences were classified using the bacterial RDP classifier in the SILVA database. The “Picante” package in R (v.4.2.0) was used to calculate the phylogenetic α diversity index used for the community, including MPD (Mean phylogenetic distance), MNTD (Mean nearest taxon distance), NRI (Net relatedness index), and NTI (Nearest taxon index). The Null model analysis was carried out using the framework described by Stegen et al., (2013), to classify community pairs into underlying drivers of deterministic process and stochastic process based on the β-nearest taxon index (βNTI) (Liu et al., 2020).

For metagenomic sequences, low-quality sequences were filtered first (mass fraction ≤38; N > 10 bp; overlap length > 15 bp). The sequences were then assembled using the MEGAHIT program,^2^ and contigs sequences over 300 bp in length were retained for subsequent studies. For the assembled metagenomic data, MetaGeneMark v.2.10 was used to classify and predict open reading frames (ORFs), and CD-HIT v.4.5.8 was used to construct non-redundant gene sets based on 90% nucleotide similarity. SOAPaligner v.2.21 for non-redundant gene set alignment. Gene abundances were obtained from reads per kilobase per million maps (RPKM values). The 16S rRNA sequence and the metagene sequence were uploaded to the NCBI database (project number PRJNA 755846).

### 2.4 Statistical analysis

One-way analysis of variance (ANOVA) was used to compare the temporal and spatial variations of SIN in soil samples. MaAslin (Multivariate Association with Linear Models) was used to analyze the relationship between dominant phylogenetic clades in bacterial communities and SIN content. The random forest model and Spearman correlation analysis were used to investigate the relationship between phylogenetic α diversity and SIN variations. The relationship between the β-NTI matrix, functional gene similarity matrix, and similarity of the SIN content was verified based on the Mantel test. The linear fitting between the β-NTI index and SIN content in each estuary was carried out to verify the effect of SIN content on phylogenetic turnover. Network models were constructed by Networkx software in Python, and OTUs with top 100 relative abundances were selected. The average path length (APL), network transitivity (Tarn), and network diameter (ND) were selected to characterize the network complexity. Then the relationship between SIN content and the above network topological values was examined based on the random forest model, Spearman correlation analysis, and Mantel test. Student’s t-test was used to examine the temporal and spatial differences between functional genes and metabolic pathways of the N cycle and linear regression analysis was used to verify the relationship between functional genes dissimilarity and ammonia nitrogen content. Structural equation modeling (SEM) was used to explore the direct and indirect relationships among all variables in the model, performed using the “lavaan” package in R (v4.2.3; http://www.r-project.org/). Considering the obvious spatiotemporal variations of SIN in environmental factors, temperature was selected as the representative of the environmental gradient. Principal component analysis (PCA) was used to reduce the dimension of variables of the nitrogen cycle function genes before SEM analysis. To demonstrate and illustrate the importance and influence of microbial community on nitrogen transformation in estuarine sediments, two structural models including environmental factors and phylogenetic diversity were simulated and compared.

## 3 Result

### 3.1 Spatial variation in SIN, and links with soil properties and bacterial biodiversity

The one-way ANOVA results showed that there were no significant variations between high-latitude regions and low-latitude regions in SIN concentrations (one-way ANOVA, *p* > 0.05). However, obvious seasonal variations in SIN concentrations were found in the estuarine coastal zone of China. In all estuarine areas, concentrations of soil nitrite nitrogen (NO_2_^−^-N) and ammonium nitrogen (NH_4_^+^-N) in September were significantly lower than that in March (one-way ANOVA, *p* < 0.05). While the nitrate nitrogen (NO_3_^−^-N) concentration was significantly higher in September than that in March (one-way ANOVA, *p* < 0.05).

The results of the MaAslin analysis showed that the bacterial phylogenetic clades affected by SIN were significantly different in different seasons (Supplementary Table S2). For example, in the samples from March, the relative abundance of *Bacteroidetes* was significantly associated with NO_3_^−^ and NO_2_^−^ content. However, it was significantly correlated with the concentration of NH_4_^+^ in September. Moreover, the number of bacterial clades abundance significantly correlated with SIN concentration in March was significantly higher than that in September, suggesting that the concentration of SIN might affected the seasonal variation of bacterial composition of dominant clades. The correlation analysis showed that the correlation between SIN and the α-diversity index of the bacterial community was obvious seasonal variation. For example, NH_4_^+^ was significantly and negatively correlated with the Sobs, Ace, and Chao 1 index, while in September, it was the opposite (Supplementary Figure S1).

### 3.2 Relationship between bacteria phylogenetic diversity, network relationship, phylogenetic turnover, and SIN variation

**Figure 1:**
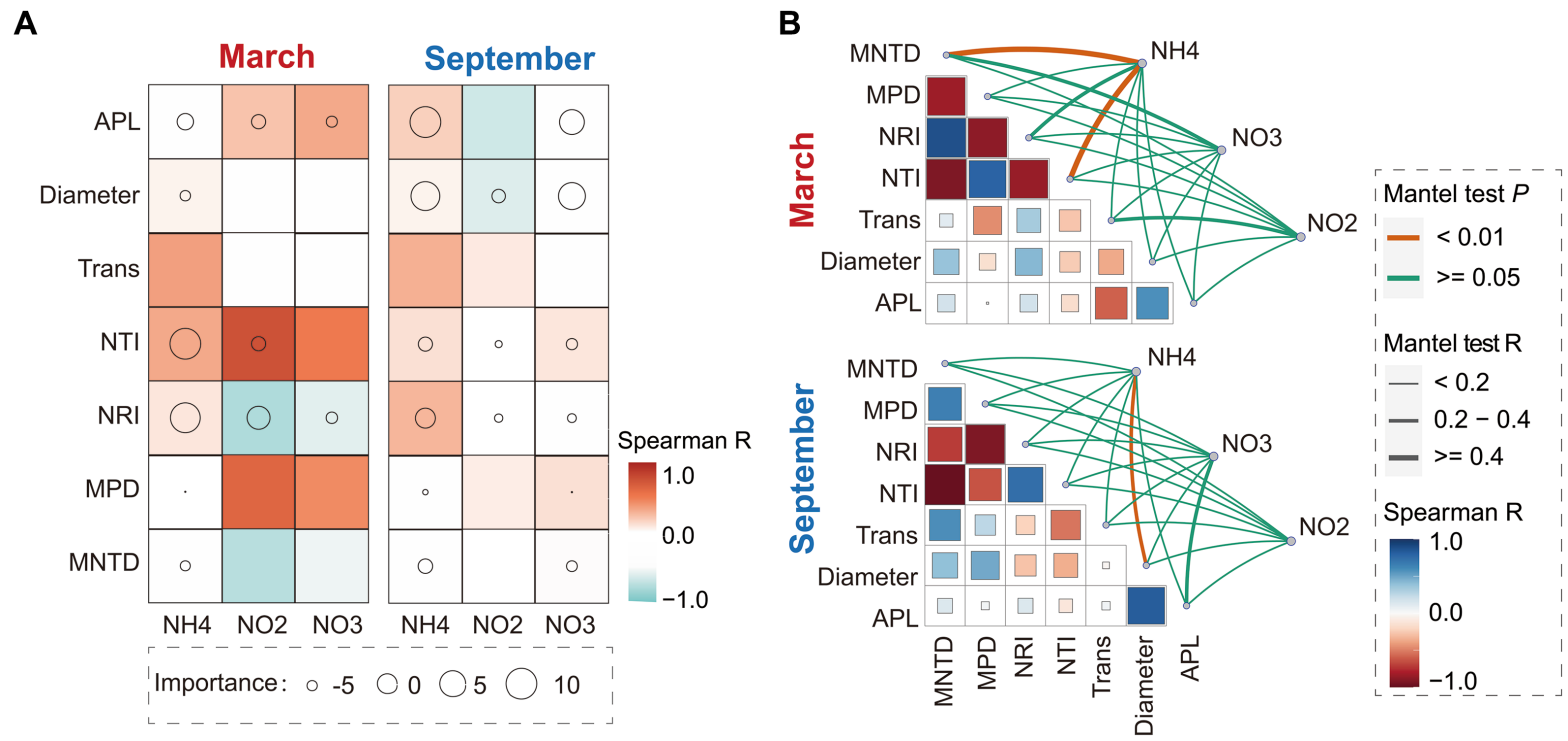
Random forest and Spearman correlation analysis between the soil inorganic nitrogen (SIN) content, network topological properties, and phylogenetic diversity characteristics in March and September **(A)**. Circle size indicates SIN importance, while color represents Spearman correlation. Additionally, the Mantel test and Spearman correlation analysis were conducted to examine the association between network topological properties, phylogenetic diversity characteristics, and SIN content **(B)**. The thickness of lines reflects the strength of correlation, with color indicating Spearman correlation. NO2, nitrite nitrogen; NO3, nitrate nitrogen; NH4, ammonium nitrogen. APL, average path length; Tarn, transitivity; MPD, mean phylogenetic distance; MNTD, mean nearest taxon distance; NRI, net relatedness index; NTI, nearest taxon index.

The results of statistical analysis revealed significant seasonal variations in the associations between phylogenetic diversity and occurrence patterns of bacterial communities with SIN in China’s estuarine intertidal zone. In March, the random forest analysis demonstrated that soil NH_4_^+^-N and NO_2_^−^-N contents critically regulated the phylogenetic diversity indices (e.g., NTI and NRI indices) of bacterial communities (Figure 1A). Mantel test results indicated a significant correlation between MNDT and NTI indices with soil NH_4_^+^-N content (*p* < 0.01, *R*_mantel_ > 0.4) (Figure 1B).

However, in September, Soil NH_4_^+^-N and NO_3_^−^-N contents emerged as key factors in SIN influencing the characteristics of bacterial co-occurrence network, including APL and network diameter (the importance index; MSE% > 10) (Figure 1A). In addition, the Mantel test results exhibited a strong correlation between soil NH_4_^+^-N and NO_3_^−^-N contents with APL index and network diameter (*R*_mantel_ > 0.4), while the NO_3_^−^-N content was not significantly associated with APL (*p* > 0.05) (Figure 1B). Additionally, the results show that the bacterial phylogenetic diversity in March exhibits a conspicuous correlation with soil SIN content, implying that the characteristics of NH_4_^+^-N content play a pivotal regulatory role in the community evolution of the bacteria. In September, while phylogenetic indices of bacterial communities show a weak correlation with SIN content, the ecological associations in bacterial communities such as co-occurrence patterns demonstrate a strong correlation with soil NH_4_^+^-N and NO_3_^−^-N content.

**Figure 2:**
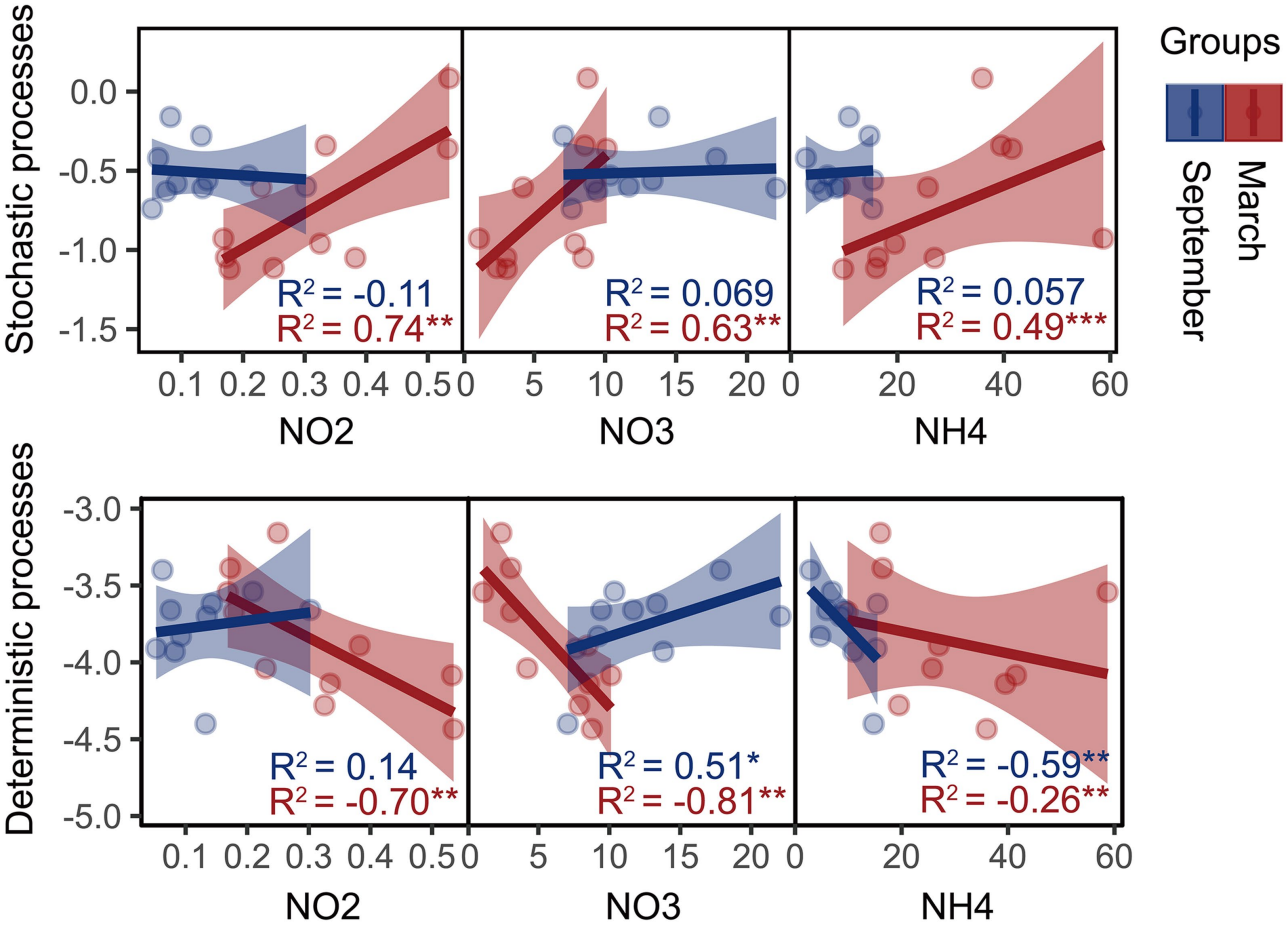
Linear least-squares regression analysis was used to correlate soil inorganic nitrogen (SIN) content with the microbial community assembly processes in March and September. Red and blue represent samples from March and September, respectively, with asterisks indicating significance levels: *p < 0.05; **p < 0.01; ***p < 0.001. NO2, nitrite nitrogen; NO3, nitrate nitrogen; NH4, ammonium nitrogen.

Moreover, we founded on the utilization of the β-NTI index to unveil the phylogenetic turnover in bacterial community systems and investigate their potential correlation with variations in SIN content. Mantel results demonstrate a significant correlation between the β-NTI index and the NH_4_^+^-N, NO_2_^−^-N, and NO_3_^−^-N content (*p* < 0.01, *R*_mantel_ > 0.4), indicating that SIN plays a crucial role in shaping the phylogenetic turnover of estuarine intertidal bacteria. Furthermore, the linear regression analysis revealed a significant correlation between the assembly processes of the bacterial community and SIN content in March (Figure 2). In September, the deterministic process in the bacterial community was significantly regulated by NH_4_^+^-N and NO_3_^−^-N content.

### 3.3 Functional characteristics of nitrogen metabolism, and links with SIN content

**Figure 3:**
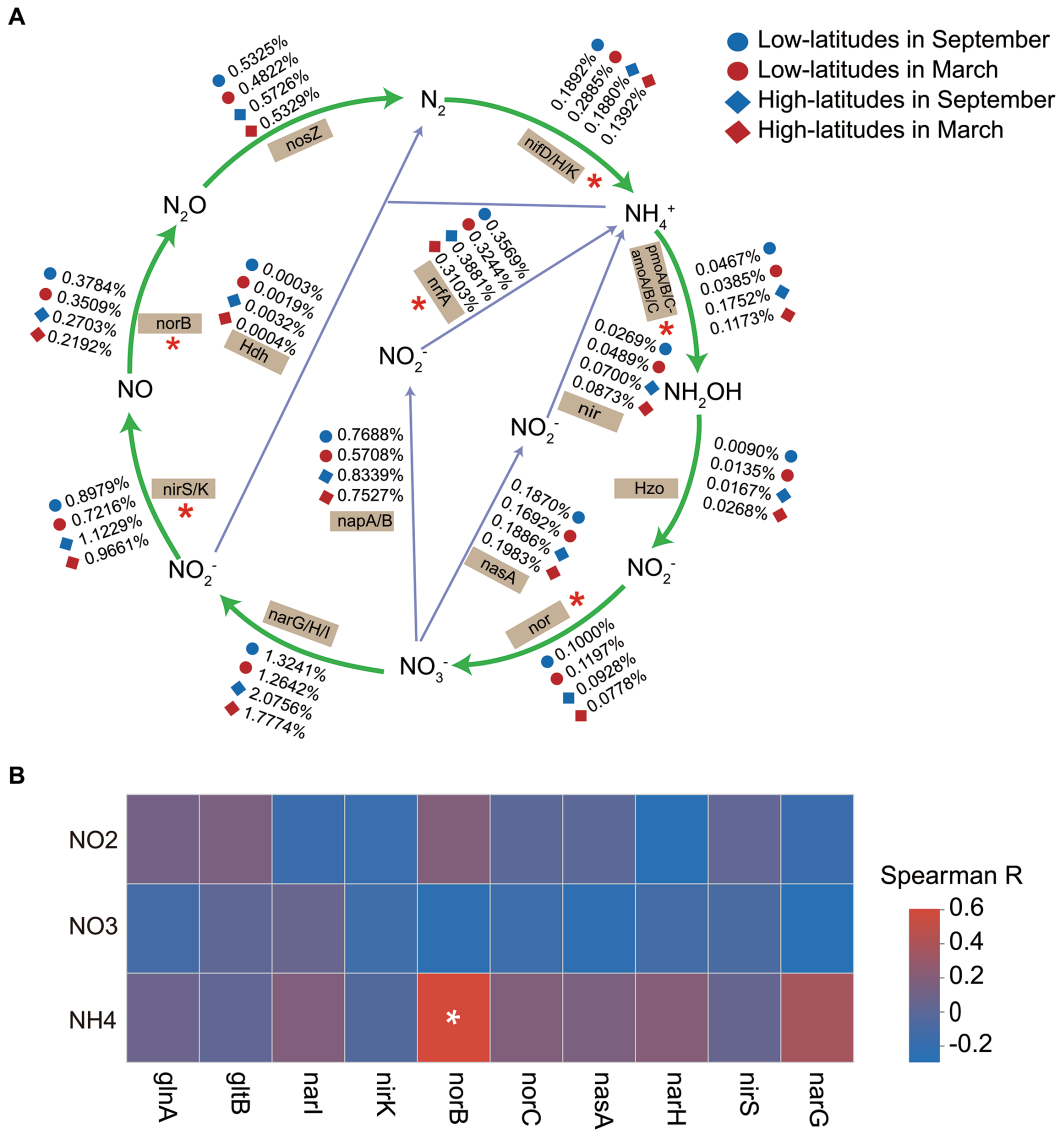
The temporal and spatial variations in soil microbial nitrogen metabolism **(A)**, with significant spatiotemporal differences in the relative abundance of functional genes indicated by asterisks (*). Spearman correlation heat map depicts the relationship between the relative abundance of nitrogen cycling functional genes and soil inorganic nitrogen (SIN) content **(B)**, with color representing Spearman correlation values (*p < 0.05). NO2, nitrite nitrogen; NO3, nitrate nitrogen; NH4, ammonium nitrogen.

The relative abundances of *nif* genes (*nifD*/*H*/*K*) for nitrogen fixation, the *amoA*/*B*/*C* genes of the nitrification pathway, *nir* and *nor* genes of the denitrification pathway, and the identification of functional genes (*nrfA* gene and *hdh* gene) responsible for driving dissimilatory nitrate reduction to ammonium (DNRA) showed significant spatiotemporal heterogeneity (Figure 3A). Additionally, the Mantel test results revealed a significant correlation (*p* = 0.003 and 0.006 in March and September, respectively) between the spatial dissimilarity of functional genes associated with the nitrogen metabolism and the SIN contents. Suggesting that variations in sediment SIN content impact the distribution patterns of the functional potential of the nitrogen cycle. Implied that the variations of SIN in soil may have an impact on the nitrogen cycling process in soil microorganisms. However, the linear regression model was utilized to investigate the potential correlation between SIN concentration and the functional capacity of nitrogen-cycling genes, and there was no significant association between SIN concentration and genes involved in nitrogen cycling (*p* > 0.05). Similarly, The Spearman correlation analysis revealed a weak association between the relative abundance of the top 10 nitrogen metabolism function genes and SIN content (Figure 3B). This indicates that, within the scope of this study, there is insufficient evidence to assert a strong influence of SIN on these genes.

### 3.4 Structural equation modeling (SEM) analysis

**Figure 4:**
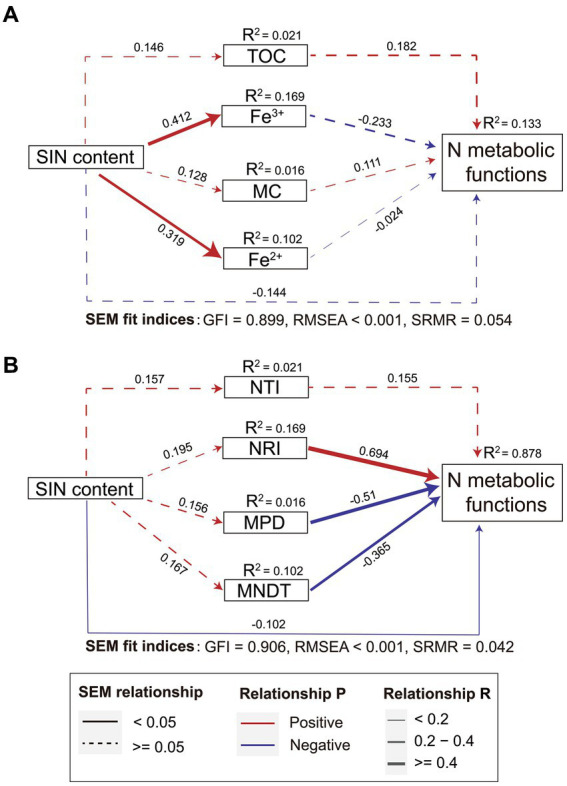
The direct and indirect effects of soil inorganic nitrogen (SIN) content, environmental factors **(A)**, and microbial phylogenetic diversity **(B)** on microbial nitrogen metabolism were investigated based on the structural equation modeling (SEM) model. The red and blue lines represent positive and negative correlations respectively, the thickness of the line segment indicates the strength of the correlation, and the solid line indicates the significance of the correlation (p < 0.05). NTI, nearest taxon index; NRI, net relatedness index; MPD, mean phylogenetic distance; MNTD, mean nearest taxon distance.

SEM analysis was used to explore the direct and indirect effects of environmental factors, microbial phylogenetic α diversity, and SIN content on the functional potential of the nitrogen cycle. Although the association is not significant, SEM showed the SIN directly affects the nitrogen metabolism function. Additionally, the nitrogen metabolism function is also affected indirectly by the influence of iron ion concentration. However, the model including microbial phylogenetic diversity significantly improved the predictability of nitrogen cycle function in estuarine soils. The total variance of the nitrogen cycle function explained by the fixed factor increased by 6.6 times, based on R^2^ values. The results depicted in Figure 4 indicate that the soil physicochemical properties (Figure 4A) did not exhibit a statistically significant impact on the functional potential of bacterial nitrogen cycling when compared to models incorporating only microbial community phylogenetic diversity (Figure 4B). In summary, these contrasting models underscore the potential contribution of phylogenetic characteristics within bacterial microbial communities towards nitrogen transformation in estuarine ecosystems.

## 4 Discussion

This study focused on the relationship between phylogenetic diversity, nitrogen-cycling functional profiles, and SIN concentration in estuarine soils. Our results showed that the phylogenetic α diversity and phylogenetic turnover (known as phylogenetic β diversity) were affected by SIN concentration in sediments, and the nitrogen-cycling functional profiles of bacterial communities were more obviously regulated by the microbial phylogenetic diversity.

The key to revealing the nitrogen metabolism driven by microorganisms lies in understanding the response mechanism of microbial physiological, ecological, and phylogenetic characteristics to SIN content. The findings of this study demonstrate significant seasonal differences in microbial communities related to SIN concentration at the bacterial taxonomic changes, which reflects the screening effect of habitat environmental characteristics on species selection. SIN plays a crucial role in determining the distribution of microorganisms in different seasons. Previous studies have reported that seasonal variations in SIN concentration in aquatic and sediment environments can influence microbial community composition (Hou et al., 2015; Bao et al., 2016). Microorganisms involved in nitrogen cycling are influenced not only by substrate availability but also by other environmental factors such as pH, salinity, redox potential, etc., which affect their growth and reproduction. Furthermore, different bacteria exhibit varying affinities for ammonia or nitrogen, which is a major factor influencing how SIN concentration affects microbial community structure (Ward et al., 2011). Additionally, within local or regional habitats, microbial communities exhibit complex interrelationships including competition, collaboration, mutualism, etc. Although this intricate network relationship demonstrates overall stability; it also allows for turnover or changes in microbial community structure (Wertz et al., 2012). In short, the effects of SIN concentration on microbial communities within natural environmental systems are multifaceted with both direct and indirect impacts. Herein, our study confirms that similar to previous research findings; seasonal fluctuations in sediment SIN concentration impact the ecological and evolutionary characteristics of estuarine bacterial communities.

The findings of our study demonstrate that variations in soil SIN concentrations, particularly NO_2_^−^ and NH_4_^+^, exert a significant impact on the diversity, richness, and phylogenetic characteristics of bacterial communities. Notably, these effects are more pronounced during March compared to September. Furthermore, our study revealed a significant influence of soil SIN concentration on bacterial assembly processes, with changes in SIN concentration dictating the assembly of bacterial communities. For instance, high SIN environments tend to be dominated by random processes in September, whereas deterministic processes tend to predominate in low SIN concentration soils. The response of microorganisms to environmental changes and ecological preferences is manifested in group behavior, indicating a preference for specific environmental characteristics or the environmental gradient for the microbial groups (Hubbell, 2001; Martiny et al., 2009; Martiny et al., 2015). This novel perspective offers insights into explaining variations in microbial composition and differences in microbial biogeographical distribution, thereby facilitating predictions regarding the evolutionary adaptation characteristics of microorganisms to changing environments (Aguirre De Cárcer, 2019).

For example, the geographical distribution of estuaries’ bacterial communities is primarily influenced by pH or salinity. Bacterial preference for salinity can be categorized into freshwater and marine species, and the microbial communities exhibit highly conservative response characteristics to changes in pH (Bahram et al., 2018). Consequently, the ecological and evolutionary responses in bacteria groups to SIN variation suggest a potential regulatory role of inorganic nitrogen content on microorganisms. Studies conducted in this region, as indicated by Zhang et al., (2023), have shown that the dominance of selectivity processes mediated by environmental factors was unimportant. However, it is crucial to acknowledge that the response of microorganisms to SIN, as observed in this study, can significantly impact their ecological assembly. This holds significant implications for their ecological functions and the mitigation of sediment nitrogen pollution.

The results of this study indicate that there is no significant temporal and spatial difference in the relative abundance of nitrogen cycling genes in estuarine sediments, and the correlation with SIN concentration is weak. This may be attributed to the high functional redundancy of bacterial communities in estuarine soils (Louca et al., 2018). The SEM analysis elucidated both direct and indirect effects on the functional potential of the nitrogen cycle. In this model, the initial variable represents spatiotemporal variation in inorganic nitrogen content in estuarine soils. Subsequently, the environmental parameters further influenced the nitrogen-cycling potential in soil microbial communities. Indeed, changes in SIN also impact the phylogenetic characteristics of microbial communities. Consistent with previous studies (Li et al., 2021), within the SEM model, environmental factors (TOC, MC, and iron ion concentration) have indirect and insignificant effects on bacterial nitrogen cycling potential if indicators of microbial evolutional characteristics were not included. However, when considering bacterial phylogenetic characteristics within the SEM model, a direct and significant effect on nitrogen metabolism functional potential emerges with a regression coefficient that increases by 6.6 times. These findings demonstrate a potential relationship between microbial community phylogenetic characteristics and soil nitrogen metabolism.

The estuarine intertidal zone is a key site to alleviate the input of high-load nitrogen from land into the ocean. Soil bacterial communities are the dominant biological groups mediating soil nitrogen removal. Herein, we focused on the potential effects of variation in SIN content on bacterial community ecology, phylogenetic characteristics, and ecological functional potential of nitrogen metabolism. The results showed that bacterial community characteristics including diversity, co-occurrence patterns, and phylogenetic characteristics were significantly influenced by SIN spatial and temporal heterogeneity. However, the nitrogen metabolism of bacterial communities was related to their phylogenetic characteristics but not directly regulated by SIN properties, which might be due to the high degree of functional redundancy within estuarial bacterial communities.

In conclusion, this study highlights that estuarine SIN content directly affects changes in the phylogenetic characteristics of soil microorganisms and is weakly associated with bacterial nitrogen metabolic potential. However, field investigations alone cannot fully comprehend the environmental mechanisms underlying the ecology, evolution, and functional characteristics of estuarine microbial communities. Additionally, the temporal and spatial variations of ecological, evolutionary, and ecological functional traits in these bacterial communities are influenced by multiple complex environmental factors. Nevertheless, the findings provide valuable insights into the ecological, evolutionary, and functional responses of estuarine bacterial communities to SIN content. These insights are crucial for understanding both the mechanisms driving the geographical distribution of soil microorganisms and the responses of soil nitrogen cycling to changes in nitrogen input in estuarine soils.

## Data availability statement

The original contributions presented in the study are included in the article/Supplementary material, further inquiries can be directed to the corresponding author.

## Author contributions

SL: Investigation, Methodology, Supervision, Writing – original draft. TL: Data curation, Writing – review & editing. CL: Software, Writing – review & editing, Validation. DS: Software, Writing – review & editing. QY: Data curation, Writing – review & editing, Software. DG: Writing – review & editing, Formal analysis, Methodology, Visualization. ZZ: Writing – review & editing, Conceptualization, Project administration, Resources.

## Footnotes

^1^ www.worldclimate.org

^2^ https://github.com/voutcn/megahit
