# Supplementary material for: Impact of soil inorganic nitrogen on bacterial phylogeny in estuarine intertidal zones: a study of nitrogen metabolism
Source: Front Microbiol. 2024 Jan 5;14:1341564. doi: 10.3389/fmicb.2023.1341564 (PMC10797050; doi:10.3389/fmicb.2023.1341564)
Supplement: Supplementary file 1 [file Data_Sheet_1.docx]

**Supplementary information**

**Impact of Soil Inorganic Nitrogen on Bacterial Phylogeny in Estuarine Intertidal Zones: A Study of Nitrogen Metabolism**

Siqi Li ^a^, Tianyang Liu ^a^, Cheng Liu ^b^, Donglei Sun ^a^, Qin Yan ^a^, Dengzhou Gao ^c^, Zongxiao Zhang ^d^^[[1]](#footnote-1)^*

^a^ Department of Military Oceanography and Hydrography & Cartography, Dalian Naval Academy, Dalian 116018, China

^b^ Shandong Key Laboratory of Eco-Environmental Science for the Yellow River Delta, Binzhou University, Binzhou, Shandong, 256600, China

^c^ Key Laboratory of Humid Subtropical Eco-geographical Process of Ministry of Education, College of Geographical Sciences, Fujian Normal University, Fuzhou 350117, China

^d^ School of Environmental Science and Engineering, Southern University of Science and Technology, Shenzhen, Guangdong, 518055, China

**Supplementary Table S1.** Sampling site information of samples in this study

| Sensons | Sampling site (number) | Latitude(°N) | Longitude(°E) |
| --- | --- | --- | --- |
| **March** |  |  |  |
|  | S1(3) | 40°47'N-40°54'N | 121°56'E-121°49'E |
|  | S2(4) | 38°46'N-39°50'N | 117°34'E-117°50'E |
|  | S3(5) | 37°34'N-38°14'N | 118°58'E-119°10'E |
|  | S4(3) | 33°46'N-33°53'N | 120°26'E-120°31'E |
|  | S5(5) | 30°06'N-31°42'N | 121°09'E-121°56'E |
|  | S6(5) | 27°56'N-28°00'N | 120°44'E-120°58'E |
|  | S7(3) | 26°03'N-26°33'N | 119°31'E-119°36'E |
|  | S8(4) | 24°23'N-24°28'N | 117°56'E-118°01'E |
|  | S9(5) | 22°08'N-22°35'N | 113°02'E-113°36'E |
|  | S10(3) | 21°35'N-21°37'N | 108°18'E-109°04'E |
| **September** |  |  |  |
|  | S1(3) | 40°48'N-40°54'N | 121°49'E-121°56'E |
|  | S2(4) | 38°46'N-39°50'N | 117°34'E-117°50'E |
|  | S3(4) | 37°49'N-38°51'N | 118°12'E-118°59'E |
|  | S4(3) | 32°01'N-33°28'N | 120°18'E-120°36'E |
|  | S5(4) | 30°16'N-31°22'N | 121°21'E-121°58'E |
|  | S6(5) | 27°49'N-28°13'N | 120°44'E-120°51'E |
|  | S7(5) | 26°03'N-26°55'N | 119°03'E-119°31'E |
|  | S8(5) | 24°27'N-24°46'N | 117°35'E-118°42'E |
|  | S9(4) | 22°08'N-22°44'N | 113°00'E-113°46'E |
|  | S10(3) | 21°41'N-21°44'N | 108°19'E-109°22'E |

**Supplementary Table S2.** MaAslin analyses of bacterial phylum abundance and sediment inorganic nitrogen concentration, taxon’s that were significantly associated with variables were shown

| Season | Variable | Feature | Coefficient | P-value |  | Season | Variable | Feature | Coefficient | p-value |
| --- | --- | --- | --- | --- | --- | --- | --- | --- | --- | --- |
| March | NH_4_^+^ | Armatimonadetes | -0.00062 | 0.007411 |  | September | NH_4_^+^ | Bacteroidetes | 0.00590 | 0.015530 |
|  |  | Dadabacteria | -0.00123 | 0.010373 |  |  |  | WS2 | 0.00057 | 0.026156 |
|  |  | Elusimicrobia | -0.00025 | 0.015357 |  |  |  | Hydrogenedentes | 0.00051 | 0.027477 |
|  |  | Fusobacteria | 0.00040 | 0.029454 |  |  |  |  |  |  |
|  |  | Fibrobacteres | 0.00016 | 0.033271 |  |  |  |  |  |  |
|  | NO_2_^-^ | Bacteroidetes | 0.48941 | 0.000254 |  |  | NO_2_^-^ | Atribacteria | 0.06527 | 0.004372 |
|  |  | Proteobacteria | 0.24914 | 0.0008 |  |  |  | Kiritimatiellaeota | 0.10409 | 0.026001 |
|  |  | Kiritimatiellaeota | 0.05234 | 0.003751 |  |  |  | Fibrobacteres | -0.08881 | 0.027661 |
|  |  | Modulibacteria | 0.01945 | 0.010014 |  |  |  | Aegiribacteria | 0.05429 | 0.029223 |
|  |  | Armatimonadetes | -0.04702 | 0.01021 |  |  |  | Dadabacteria | 0.09449 | 0.029276 |
|  |  | Fusobacteria | 0.03302 | 0.022994 |  |  |  | Nitrospirae | -0.56416 | 0.040271 |
|  |  | Actinobacteria | -0.23670 | 0.028916 |  |  |  |  |  |  |
|  |  | Acidobacteria | -0.15680 | 0.029154 |  |  |  |  |  |  |
|  | NO_3_^-^ | Armatimonadetes | -0.00251 | 0.004826 |  |  | NO_3_^-^ | Nitrospirae | 0.00805 | 0.000463 |
|  |  | Bacteroidetes | 0.01838 | 0.007143 |  |  |  | WS2 | -0.00116 | 0.000503 |
|  |  | Dependentiae | -0.00148 | 0.01502 |  |  |  | Nitrospinae | 0.00199 | 0.001513 |
|  |  | Acidobacteria | -0.00746 | 0.034866 |  |  |  | Planctomycetes | 0.00219 | 0.008385 |
|  |  | Proteobacteria | 0.00801 | 0.035772 |  |  |  | Omnitrophicaeota | 0.00039 | 0.047885 |
|  |  | Modulibacteria | 0.00076 | 0.043219 |  |  |  |  |  |  |
|  |  | Elusimicrobia | -0.00081 | 0.04706 |  |  |  |  |  |  |

**Supplementary Figure S1.**
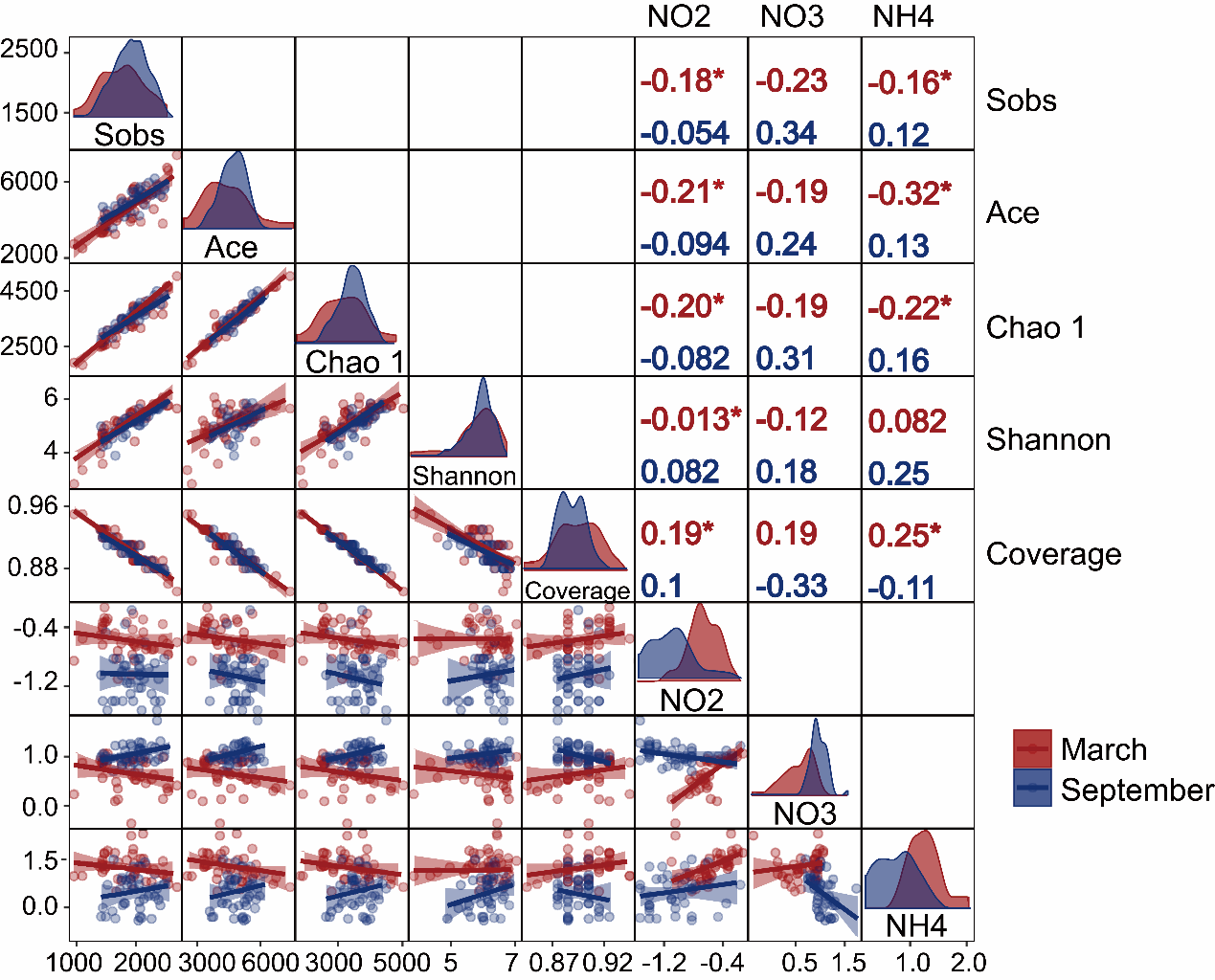
 Spearman correlation between soil inorganic nitrogen content and microbial α-diversity characteristics. * P < 0.05. NO2, nitrite nitrogen; NO3, nitrate nitrogen; NH4, ammonium nitrogen.

1. * Corresponding author: *E-mail address*: zhangzx@sustech.edu.cn [↑](#footnote-ref-1)
